# Standard Errors for Reliability Coefficients

**DOI:** 10.1017/psy.2025.10050

**Published:** 2025-09-30

**Authors:** L. Andries van der Ark

**Affiliations:** Research Institute of Child Development and Education, University of Amsterdam, Amsterdam, Netherlands

**Keywords:** Multinomial sampling, reliability analysis, reliability coefficients, standard errors, statistical software

## Abstract

Reliability analysis is one of the most conducted analyses in applied psychometrics. It entails the assessment of reliability of both item scores and scale scores using coefficients that estimate the reliability (e.g., Cronbach’s alpha), measurement precision (e.g., estimated standard error of measurement), or the contribution of individual items to the reliability (e.g., corrected item-total correlations). Most statistical software packages used in social and behavioral sciences offer these reliability coefficients, whereas standard errors are generally unavailable, which is a bit ironic for coefficients about measurement precision. This article provides analytic nonparametric standard errors for coefficients used in reliability analysis. As most scores used in behavioral sciences are discrete, standard errors are derived under the relatively unrestrictive multinomial sampling scheme. Tedious derivations are presented in appendices, and R functions for computing standard errors are available from the Open Science Framework. Bias and variance of standard errors, and coverage of the corresponding Wald-based confidence intervals are studied using simulated item scores. Bias and variance, and coverage are generally satisfactory for larger sample sizes, and parameter values are not close to the boundary of the parameter space.

## 1 Introduction

Reliability analysis is one of the most conducted analyses in psychology and education. For example, Cronbach’s (1951) original paper introducing coefficient alpha (Cronbach’s alpha), arguably the most popular reliability estimate, has been cited over 70,000 times.^1^ Reliability analysis can be classified into at least two approaches. The first one involves conducting reliability analysis under a measurement model that places restrictions on data, such as a factor model or an item response theory model. In this case, reliability estimates are derived from model parameters. For example, coefficient omega (McDonald, 1999; also, see Zinbarg et al., 2005) is an unbiased test-score reliability estimate under the hierarchical factor model, and the test-score reliability coefficient and latent-trait reliability coefficient derived by Andersson et al. (2022) are unbiased reliability estimates under the multigroup item response theory model. The second approach entails reliability analysis within classical test theory (e.g., Lord & Novick, 1968), a model that does not impose any restrictions on test data. For example, Lord and Novick (1968, Theorem 4.4.3) proved that Cronbach’s alpha is a lower bound to reliability under any model. This paper considers reliability coefficients under classical test theory (see, e.g., Sijtsma et al., 2024, for motivations for using classical test theory).

Reliability analysis under the classical test theory model is offered in general data analysis packages such as JASP (Love et al., 2019; JASP, 2024), SAS (SAS Institute Inc., 2023), SPSS (IBM Corp., 2021), and Stata (StataCorp., 2023), and also in R packages, such as psych (Revelle, 2024). The output of these software packages include five types of descriptive statistics (Table 1, columns 2–6): (i) *item statistics*, such as item means and item standard deviations; (ii) *scale statistics*, such as scale mean, scale standard deviation, and reliability estimators such as Cronbach’s alpha, Guttman’s lambda series (Guttman, 1945), and the split-half reliability coefficient (Guilford, 1936, p. 419); (iii) *rest statistics* (i.e., scale statistics with one item deleted), such as Cronbach’s alpha if an item were deleted; (iv) *inter-item statistics* such as correlations and covariances; and (v) *item-rest statistics*, such as item-rest correlations, which is also known as the corrected item-total correlation or 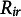 statistic. Sometimes, the item-total correlation or 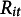 statistic is also provided. In this article, the term *reliability coefficient* is used as a generic label for all statistics reported in a reliability analysis.

**Table 1:** Reliability coefficients, number of coefficients produced per analysis for each type of coefficient, and corresponding appendix for SE derivations

Coefficient	Type					App.
	Item	Scale	Rest	Item-item	Item-rest	
Mean	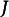	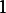	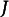			A
Variance	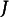	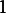	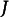			
Standard deviation	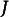	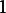	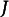			C
Covariance				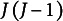	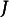	B
Correlation				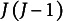	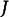	D
Covariance matrix^a^				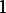		E
Lambda 1 ( 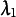 )		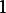	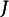			F
Lambda 2 ( 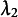 )		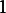	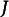			G
Alpha ( 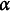 or 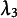 )		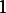	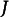			
Split-half reliability		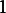	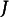			H

*Note:* Column 1 lists the reliability coefficients for which SEs were derived. Columns 2–6 indicate for six types of coefficients the number of coefficients produced in a single reliability analysis. Column 7 refers to the appendix where SE derivations are provided. 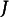 = number of items. App. = appendix.

a Covariance matrix is usually not reported, but many reliability estimators are based on inter-item covariances.

Although reliability analysis concerns precision of measurement, software providing reliability analysis generally do not provide standard errors (SEs) or confidence intervals (CIs) for the reliability coefficients. As a result, SEs or CIs are generally not reported (Oosterwijk et al., 2019). Because SEs and CIs can be used to quantify the sampling error associated with each reliability coefficient, they allow the researcher to determine whether the sample was large enough to obtain sufficiently precise estimates. For some reliability coefficients, SEs or CIs have been derived, but these SEs have generally not been included in statistical software packages. For example, Feldt et al. (1987) derived a CI for Cronbach’s alpha ( 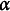 ) under the assumption that item scores satisfy the assumptions of an ANOVA model. For a 95% CI, let 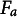 and 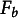 be the critical values of an *F* distribution with 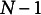 and 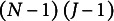 degrees of freedom, such that 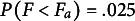 and 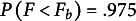 . The 95% CI is then estimated as 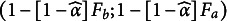 . Ahn and Fessler (2003) derived SEs for the variance and standard deviation for normally distributed scores: 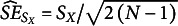 . Also, Fisher *Z*-transformation (e.g., Bartlett, 1993) normalizes the sampling distribution of the correlation coefficient, where 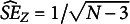 is the SE of the transformed correlation coefficient. On StackExchange (2020), answers have been provided for the SE of an estimated covariance under a bivariate normal distribution: 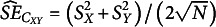 .

While most SEs are estimated under the assumption of normally distributed data, this may be unrealistic for bounded discrete scores typically used in reliability analyses, such as scores on dichotomous items. For the reliability coefficients in Table 1, this article provides estimates of SEs under multinomial sampling (see Goodman & Kruskal, 1963, for some examples), which is a relatively unrestrictive assumption suitable for discrete variables.

First, I discuss a two-step general framework for deriving SEs under a multinomial distribution, referred to as the *two-step procedure*. The two-step procedure was developed in the context of marginal modeling (e.g., Bergsma et al., 2009; Rudas & Bergsma, 2023); it is quite flexible and has been used earlier to derive SEs for relatively complicated coefficients, such as Mokken’s scalability coefficients (Kuijpers et al., 2013a) and norm statistics (Oosterhuis et al., 2017). The two-step procedure was designed for discrete data, but Oosterhuis et al. showed that it also allows the derivation of SEs for continuous scores.

Second, I discuss the SEs of the coefficients in Table 1. The derivations are often lengthy and cumbersome and are provided in appendices (see Table 1, last column). Note that Table 1 does not provide an exhaustive list of coefficients used in classical test-theory-based reliability analysis. Some coefficients that are not listed in Table 1 involve the maximum or minimum values observed in sample data (e.g., coefficients 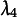 , 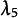 , and 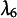 from Guttman’s, 1945, lambda series), and therefore, regularity conditions for computing first-order partial derivatives are not satisfied. Other coefficients (e.g., beta, Revelle, 1979; skewness or kurtosis) were excluded to make the task feasible, but their SEs can also be derived using the two-step procedure.

Third, I discuss the bias of the estimated SEs that was examined in a simulation study mimicking educational test data and psychological questionnaire data. Some existing estimates of SEs were included in the simulation study, serving as benchmarks. R code for estimating the SEs and conducting the simulation study, as well as the complete results of the study, is available on the Open Science Framework (Van der Ark, 2025).

## 2 A two-step procedure to derive SEs

Let 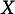 be a random variable obtained from administering a test to a simple random sample of 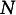 respondents, such as an item score, a sum score, or a rest score (i.e., the sum score minus the item score). For the coefficients in Table 1, the SEs of the mean, variance, and standard deviation are based on a single variable 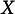 . The SEs of the covariance, correlation, and split-half reliability coefficient are based on two variables—denoted by 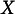 and 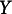 . Finally, the SEs of the lambda coefficients are based on 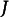 item scores—denoted by 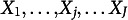 . Let 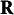 be a matrix containing all unique response patterns observed in the test administration. The response patterns are assumed to be ordered lexicographically (i.e., last column changes fastest), and the number of observed response patterns is denoted by 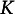 . For a dataset containing the responses of 426 respondents to 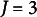 dichotomous items, Table 2 (upper panel) provides illustrations of 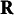 for response patterns based on a single dichotomous item score (top left), on a dichotomous item score and its rest score (top center), and on all three item scores (top left). In Table 2, all observed scores are integers, but they may also be real-valued.

**Table 2:** Three examples of response patterns collected in matrix 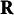 (top, for details, see note), and the corresponding frequency vectors (bottom).

One variable ( 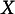 )	Two variables ( 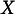 , 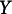 )	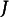 item scores ( 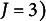
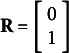	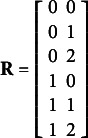	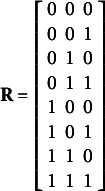
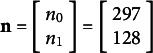	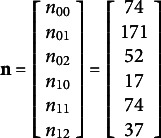	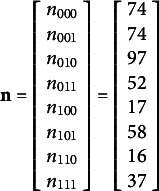

*Note*: The examples were based on a test consisting of three dichotomous items. On the left, the two response patterns (0 and 1) produced by a single dichotomous item. In the middle, the six response patterns (00, …, 12) produced by a dichotomous item and its rest score; and the eight response patterns (000, …, 111) produced by all three item scores. In this specific example, all possible response patterns are observed, which needs not be true in general.

Let 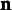 denote the vector of observed frequencies of each row in 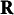 . Table 2 (lower panel) shows the vectors of observed frequencies for each of the three matrices in the upper panel with example frequencies. The subscripts of the elements of 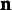 refer to the corresponding response patterns. Sometimes, it is useful to use a single index 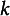 ( 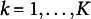 ) to indicate the ordering of elements; that is, 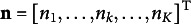 , where superscript T denotes the transpose of a matrix or vector. Let 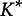 be the total number of *possible* response patterns. Under the two-step procedure, the values of 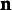 are assumed to form a random sample of size 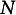 from a multinomial distribution, 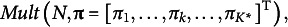 which implies that each response pattern has a positive probability of appearing in the data.

### 2.1 Step 1. Deriving the Jacobian of coefficients using the generalized exp-log notation.

Let 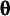 be a vector of population coefficients, with sample estimates 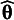 . Except for the inter-item covariance matrix, all coefficients in Table 1 are scalars, and vectors 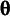 and 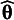 reduce to scalars 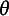 and 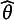 , respectively. First, it must be shown that 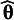 can be written as a (vector) function of 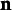 **;** that is, 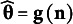 . This facilitates the computation of the Jacobian—denoted by 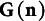 , the matrix of first partial derivatives of 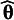 with respect to 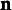 —required for deriving SEs using the delta method in Step 2. In general, deriving the Jacobian requires tedious derivations. The generalized exp-log notation developed by Grizzle et al. (1969), Forthofer and Koch (1973), Kritzer (1977), and Bergsma (1997) alleviates the burden by writing 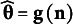 as a series of 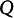 appropriate design matrices, 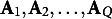 , such that
(1)

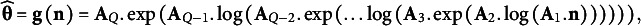

 where 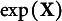 and 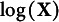 denote the exponential and natural logarithm, respectively, for each element of matrix, 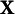 and 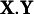 denotes the matrix product of 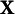 and 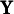 . The generalized exp-log notation facilitates the derivation of the Jacobian because the chain rule can be applied. In some cases, functions other than 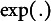 and 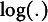 may be useful (e.g., logit, square root, or difference), hence the name generalized exp-log notation. Equation 1 can be derived using the following steps:

First, a series of 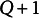 functions, 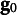 , 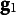 , 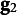 , …, 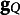 , are defined, where 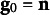 , and for 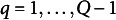 ,
(2)

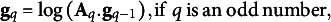

 and
(3)

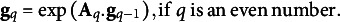



The last function is the series 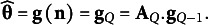 The application of the generalized exp-log notation to derive the SE of the sample mean (Appendix A) may serve as an instructive example. Appendix A also discusses the case in which 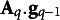 contains one or more non-positive elements, in which case 
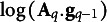
 in Equation 2 is undefined in the reals.

Next, let 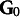 , 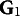 , 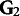 , …, 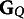 denote the Jacobians of 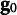 , 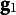 , 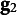 , …, 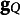 , respectively, and let 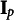 denote an identity matrix of order 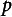 . Following standard calculus, 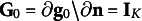 . Let 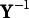 denote the inverse of 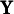 , and let 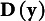 denote the diagonal matrix with vector 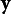 on the diagonal; then, for 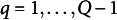 ,
(4)

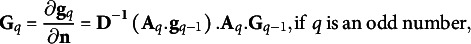

 and
(5)

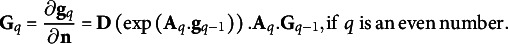



The last function in the series is 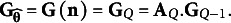 For notational convenience, in the remainder, 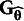 will be used as the general notation of the Jacobian of a vector of coefficients.

### 2.2 Step 2. Using the delta method to derive standard errors for reliability coefficients

If 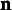 is a consistent estimator, under a multinomial sampling scheme, 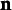 converges to its true value 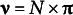 , and the central limit theorem can be applied to obtain asymptotic normality,
(6)

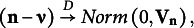

 where 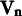 = 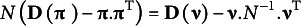 is the variance–covariance matrix of 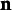 . Under a multinomial distribution, the sample estimate of 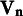 equals
(7)

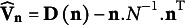

(e.g., Agresti, 2013), where 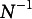 is a 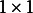 matrix with element 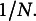 Using the first two terms of the Taylor series,
(8)

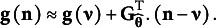



Equation 6 implies that the variance of 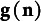 can be approximated by
(9)

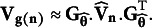



Therefore,
(10)

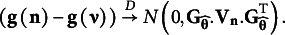



Based on Equation 10, and using the result in Equation 7, the sample estimate of the asymptotic variance of 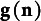 is
(11)

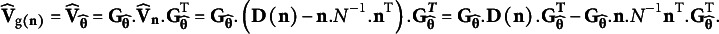



By taking the square root of the diagonal elements of 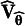 , the sample estimate of the asymptotic SE of 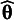 is obtained. Equation 11 can be simplified if 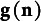 is homogeneous of order 0, which is true if 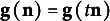 for every positive constant 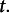 For the application here, it is useful to note that if functions are homogenous of order 0, it does not matter whether observed frequencies 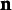 or observed probabilities 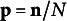 (i.e., 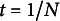 ) are used as an argument of 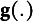 . Bergsma (1997, Appendix D) showed that if 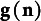 is homogeneous of order 0, then 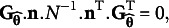 and Equation 11 reduces to
(12)

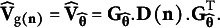



For a single coefficient (i.e., 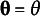 ), 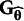 is a row vector of length 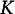 , and Equation 11 and Equation 12 can be expressed as
(13)

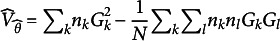

 and
(14)

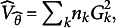

 respectively. The estimated SEs, obtained by taking the square root of the estimated variances (Equations 11, 12, 13, and 14) typically have a rather intricate form. Therefore, attempts were made to simplify the SEs to a more comprehensible form.

## 3 Large-sample estimates of the SEs of reliability coefficients

### 3.1 Mean

The estimated SE of the sample mean equals 
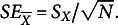
 Although this is an established result, the two-step procedure for deriving the estimated SE of the sample mean without bias correction, 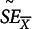 (Appendix A), is relatively simple and can serve as an instructive example of the two-step procedure.

### 3.2 Covariance

The unbiased covariance estimator equals 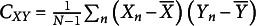 . Let
(15)

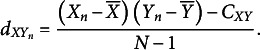



In Appendix B, it is shown that the estimated 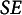 of 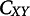 equals
(16)

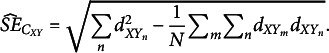



Appendix B also shows that for large 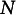 , the term 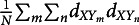 in Equation 16 tends to 0, and the estimated SE may be approximated by
(17)

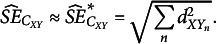



There are two special cases. First, if 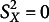 or 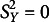 , then 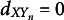 for all 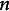 . As a result, 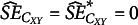 . Second, let 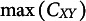 and 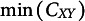 be the maximum and minimum covariance, respectively, that can be obtained given the marginal distributions of 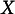 and *Y*. If 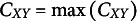 or 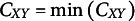 , then 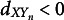 for all 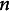 , and neither 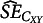 nor 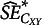 exist. These two special cases also apply to the SEs of the sample variance and the sample standard deviation in the next subsections.

### 3.3 Variance

The unbiased variance estimator equals 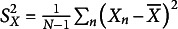 . The variance may be considered a special case of a covariance where both variables are the same; that is, 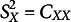 .

If 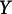 is replaced by 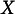 in Equation 15, Equation 15 becomes
(18)

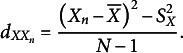



It follows directly from Equations 16 and 17 that the estimated 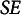 of 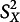 equals
(19)

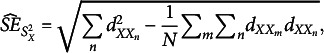

 which for large samples reduces to
(20)

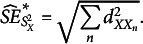



### 3.4 Standard deviation

The estimator of the standard deviation equals 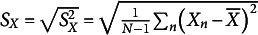 . Appendix C shows that the estimated SE of 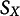 is derived by multiplying the SE of 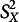 (Equations 19 and 20) by 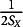 ; that is,
(21)

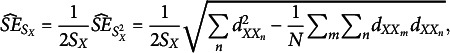

 which for large samples reduces to
(22)

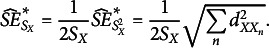



### 3.5 Correlation

The unbiased estimator of the product-moment correlation coefficient equals 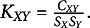 In Appendix D, it is shown that the estimated 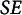 of 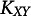 equals
(23)

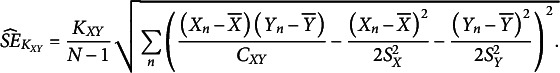



There are three special cases. First, if 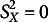 or 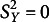 , neither 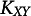 nor 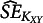 are defined. Second, if 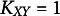 or 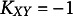 , 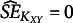 by definition. Third, let 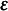 be a very small positive value. For 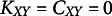 , the term 
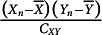
 in Equation 23 does not exist, and 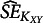 is not defined, but by replacing 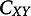 by 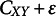 and letting 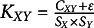 , reasonable estimates of 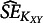 are obtained. These three special cases also hold for the split-half correlation coefficient in the next subsection.

### 3.6 Split-half reliability coefficient

Suppose the test items are split into two sets, 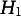 and 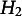 . Let 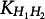 be the correlation between the sum scores on 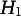 and the sum scores on 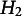 . Then, the sample value of the split-half reliability coefficient is 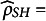 2 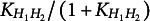 . Appendix E shows that the estimated SE of the split-half reliability coefficient equals
(24)

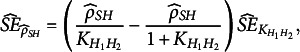

 where 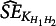 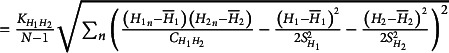 is the SE of the correlation between the two halves (cf. Equation 23).

### 3.7 Lambda coefficients

Reliability coefficients 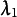 , 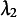 , 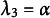 are based on the inter-item sample variance-covariance matrix 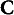 . Let 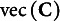 be a 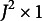 column vector obtained by stacking the column vectors of 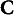 on top of one another. In Appendix F, 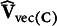 , the 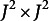 variance covariance matrix of 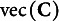 , is derived. Let 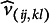 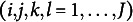 denote the elements of 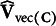 , where 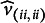 denotes the estimated variance of 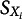 (Equation 19), 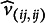 the estimated variance of 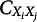 (Equation 16), 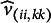 the estimated covariance of 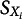 and 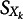 , 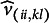 the estimated covariance of 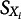 and 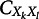 , and 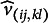 the estimated covariance of 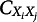 and 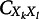 . Because 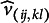 has an intricate form that could not easily be simplified, 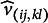 is presented only in the generalized exp-log notation (see Equation F4, F5, F6, F7, and F8 in Appendix F).

*Guttman’s* 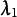 . The sample value of Guttman’s lambda 1 equals 
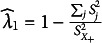
 , where 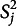 denotes the sample variance of item 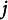 and 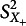 denotes the sample variance of the sum score. Let 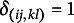 if 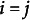 or 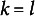 , then Appendix G shows that the estimated SE of 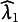 equals
(25)

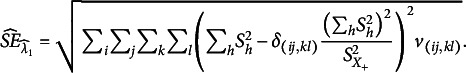



*Guttman’s* 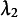 . The sample value of lambda 2 equals 
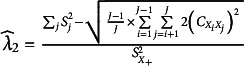
 . Let 
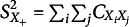
 denote the sample variance of the sum score, let 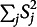 denote the sum of the sample variances of the 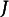 items, let 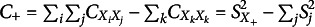 denote the sum of all sample covariances, let 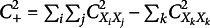 denote the sum of all squared sample covariances, and let 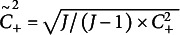 ; then, 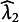 reduces to 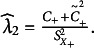 Furthermore, let 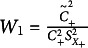 , 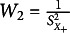 , and 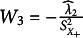 be three constant values, and let 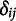 be Kronecker delta (i.e., 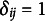 if 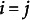 , 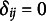 otherwise). Appendix H shows that the estimated SE of 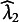 equals
(26)

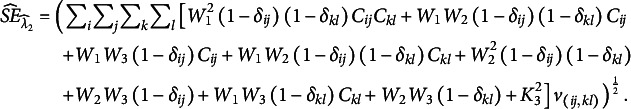



*Guttman’s* 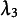 . Guttman’s 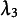 equals Cronbach’s alpha and also equals 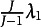 (Guttman, 1945). The SE can therefore be derived directly from 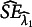 (Equation 25) as
(27)

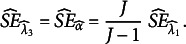



## 4 Simulation study

For different sample sizes and different types of coefficients (see Table 1), the bias of the proposed SEs and the coverage of corresponding Wald CIs for all coefficients in Table 1 were investigated using simulated data. SEs obtained by methods discussed in the Introduction section, were included as benchmarks.

### 4.1 Method

#### 4.1.1 Population model

A two-dimensional extension of Samejima’s (1995) five-parameter multidimensional acceleration model (5PAM; see, e.g., Van Abswoude et al., 2004) for dichotomous items was used as a population model. Let 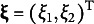 denote the vector of latent traits. For item 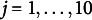 and dimension 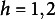 , let 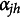 and 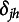 be discrimination and location parameters, respectively. Let 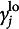 and 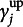 denote the lower and upper asymptotes of the item response function; 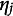 the acceleration parameter; and 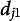 and 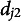 design parameters. Then, the probability of a score 1 on item 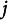 , given the latent trait vector 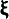 , is
(28)

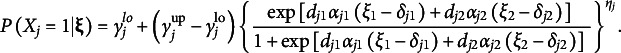



In this study, 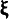 followed a bivariate standard normal distribution with correlation 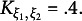 The discrimination parameters were randomly sampled from a lognormal distribution with mean 0 and standard deviation 0.1. Location parameters 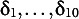 were evenly spaced in the range 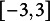 . Parameters 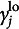 , which allow the lower asymptote to be greater than 0, were sampled from 
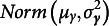
 ; parameters 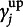 , which allow the upper asymptote to be less than 1, were sampled from 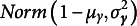 ; and parameters 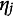 , which allow the item response function to be asymmetric, were sampled from 
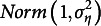
 . Parameters 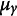 , 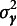 , and 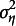 and the design parameters 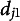 and 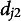 varied across the design cells (see below). Note that if 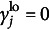 , 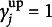 , and 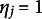 , Equation 28 reduces to the two-dimensional two-parameter logistic model. For each design cell, the population values of the reliability coefficients (*θ*) were computed from item scores—derived via Equation 28—based on a sample of 10 million 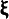 values.

#### 4.1.2 Data generation

First, 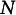 latent-trait values ( 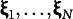 ) were sampled, and the corresponding values of 
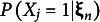
 ( 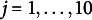 ) were computed using Equation 28. For design cells with 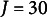 items, the probabilities were determined by setting 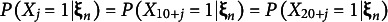 , 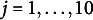 . Let 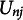 
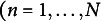
 ; 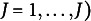 be random draws from the uniform distribution 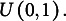 The score of respondent 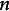 on item 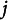 was equal to 1 if 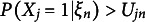 and 0 otherwise. Finally, respondent scores were collected in an 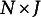 data matrix. The data generation process was replicated 2,000 times yielding datasets 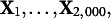 coefficients 
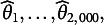
 and estimated SEs 
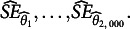


#### 4.1.3 Independent variables

***Sample size.*** Bias and coverage were investigated for three sample sizes ( 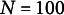 , 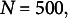 and 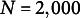 ). 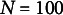 may be considered too small for reliability analyses, but this sample size was included to investigate the behavior of the SEs and CIs in relatively small samples.

***Reliability coefficients.*** All coefficients listed in Table 1 were included. It can be expected that as an estimated coefficient approaches its theoretical bound—or if one or more of the statistics on which the coefficient is based approach their bounds—the bias of the SEs will increase and the coverage of the CI will decrease. Therefore, SEs of the sample mean, sample variance, and sample standard deviation were investigated for a low-mean item (i.e., the first item, referred to as *item A* 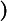 , which is relatively close to the upper bound of 1; for a high-variance item (i.e., the item in the middle, for which 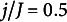 , referred to as *item B*), which is relatively close to upper bound 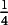 ; and for the sum score. The SEs of the sample covariance and sample correlation were investigated for item A and item B; item A and its rest score; and item B and its rest score. All other coefficients were investigated using the scores on all 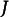 items.

***Dimensionality.*** One-dimensional and two-dimensional versions of the model in Equation 28 were investigated. In the one-dimensional model, item response depended only on 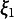 by setting 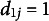 and 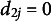 . In the two-dimensional model, odd items depended on 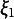 ( 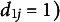 and to a lesser extent on 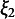 ( 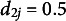 ), whereas even items depended only on 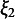 ( 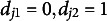 ).

***Number of items.*** The number of items was 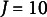 and 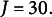


***Model complexity**.* Either the 5PAM or the 2PLM was investigated. The 5PAM was obtained by setting 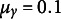 , 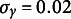 , and 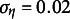 when generating item parameters. The 2PLM was obtained by setting 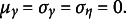


#### 4.1.4 Dependent variables

The standard deviation of 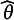 across the 2,000 replications was considered the true SE of 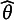 , 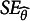 . Let 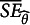 be the mean value of 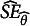 across the 2,000 replications; then, the scaled bias of 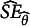 was computed as
(29)

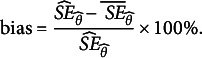



Except for the sample mean, in each replication, a 95% CI was computed using 
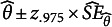
 . For the sample mean, instead of a normal deviate, a t distribution with df = 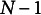 was used. The coverage was the percentage of times the CIs included the population value 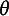 . For the conditions examining the SEs of the variance and standard deviation of item B, in some replications, the sample coefficient was on the boundary of the parameter space, and the SE did not exist. These replications were omitted from the results. To accurately interpret the values of the coverage, a 95% Agresti–Coull (1998) CI was derived, which equaled 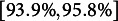 .

### 4.2 Results

The effects of dimensionality, number of items, and model complexity were small to negligible. Therefore, the results for the one-dimensional 2PLM for 10 items are reported here. The complete results are available in the supplementary material.

#### 4.2.1 Mean

The bias of the SEs of the sample mean was close to negligible (Table 3). Under conditions in the supplementary material, for 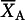 , some undercoverage was observed for 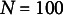 . As the population mean for this item is relatively close to the boundary, the sampling distribution is skewed to the left. For small samples, it may not be represented well by a t distribution.

**Table 3:** Bias of 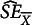 and coverage of the corresponding 95% Wald CI

Coef.	Par.	Scaled bias			Coverage		
		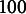	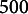	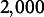	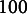	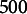	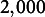
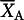	0.910	1.7%	2.1%	−0.7%	94.5**%**	**96.2%**	95.0%
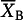	0.575	1.2%	1.1%	1.5%	94.9%	95.5%	93.9%
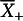	4.950	−1.3%	0.9%	3.4%	94.3%	95.2%	95.6%

*Note*: Coef. = coefficient, Par. = parameter value rounded to three decimals. Values 100, 500, and 2,000 in columns represent sample sizes. Item A is a low-mean dichotomous item, and item B is a high-variance dichotomous item (for details, see text). 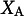 and 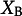 denote the scores on items A and B, respectively, and 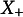 denotes the sum score. Coverage percentages outside the 95% Agresti–Coull CI [93.9%, 95.8%] are shown in boldface. Scaled bias larger than 10% is shown in boldface.

#### 4.2.2 Covariance

Under the proposed method, the SEs of the sample covariances exhibited negligible bias, with coverage at the expected level (Table 4, upper panel). Under the alternative method, bias was much larger, and none of the coverage percentages fell within the Agresti–Coull CI, indicating that the method’s assumptions were too strong for this type of data.

**Table 4:** Bias of 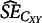 and coverage of the corresponding 95% Wald CI as estimated by the proposed method (upper panel), and under normality with homogeneous variances (StackExchange, 2020) (lower panel)

Coef.	Par.	Scaled bias			Coverage		
		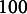	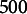	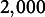	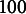	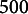	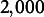
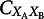	0.015	0.1%	2.8%	−1.4%	95.2%	95.7%	95.0%
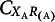	0.086	1.4%	2.1%	0.5%	**93.8%**	95.3%	95.2%
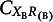	0.258	−1.3%	0.9%	3.4%	94.2%	94.7%	95.5%
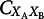	0.015	**12.4%**	**14.4%**	9.5%	**97.8%**	**97.9%**	**96.6%**
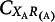	0.086	**204%**	**200%**	**194%**	**100%**	**100%**	**100%**
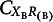	0.258	**84.6%**	**85.7%**	**88.0%**	**100%**	**99.9%**	**100%**

*Note*: Coef. = coefficient, Par. = parameter value rounded to three decimals. Values 100, 500, and 2,000 in columns represent sample sizes. Item A is a low-mean dichotomous item, and item B is a high-variance dichotomous item (for details, see text). 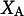 and 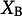 denote the scores on items A and B, respectively, and 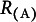 ad 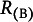 denote the rest scores of item A and B, respectively. Coverage percentages outside the 95% Agresti–Coull CI [93.9%, 95.8%] are shown in boldface. Scaled bias larger than 10% is shown in boldface.

#### 4.2.3 Variance and standard deviation

Except for differences that can be attributed to Monte Carlo error, bias variances and standard deviations were identical. Therefore, only the results for the standard deviation are reported. Under the proposed method, the bias of the estimated SEs appears to be small (Table 5, upper panel). For item B, the results indicate undercoverage of the CI. This is to be expected because 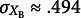 , which is very close to the theoretical upper bound .5. As a result, the sampling distribution of 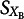 is skewed to the left (see histograms of the sampling distribution 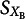 , Van der Ark, 2025), and the normal approximation does not work well, even for larger samples. For item A and the sum score, the coverage was satisfactory for 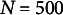 and 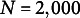 . Under the alternative estimation method (Table 5, lower panel), the bias was much larger, and none of coverage percentages were within the Agresti–Coull CI, suggesting that the method assumptions were too strong for this type of data.

**Table 5:** Bias of 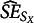 and coverage of the corresponding 95% Wald 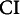 as estimated by the proposed method (upper panel), and under normality (Ahn & Fessler, 2003) (lower panel)

Coef.	Par.	Scaled bias			Coverage		
		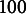	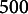	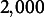	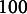	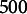	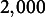
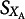	0.286	−2.0%	1.4%	−1.0%	94.8%	**96.0%**	95.0%
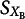	0.494	−2.4%	0.5%	1.1%	**84.1%**	**89.5%**	**93.0%**
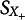	1.802	−1.7%	0.4%	0.7%	**93.4%**	95.0%	95.2%
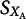	0.286	**−52.0%** ^1^	**−50.3%**	−51.3%	**71.2%**	**64.2%**	**65.7%**
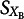	0.494	**318%**	**367%**	**370%**	**100%**	**100%**	**100%**
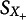	1.802	8.4%	9.6%	9.8%	**95.9%**	**96.7%**	**96.9%**

*Note*: Coef. = coefficient, Par. = parameter value rounded to three decimals. Values 100, 500, and 2,000 in columns represent sample sizes. Item A is a low-mean dichotomous item, and item B is a high-variance dichotomous item (for details, see text). 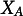 and 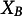 denote the scores on items A and B, respectively, and 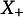 denotes the sum score. Coverage percentages outside the 95% Agresti–Coull CI [93.9%, 95.8%] are shown in boldface. Scaled bias larger than 10% is shown in boldface.

1 55 replications resulted in an NA, and results are based on 1,945 replications.

#### 4.2.4 Correlation

Under the proposed method, relative bias was large and coverage too low for 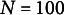 , but both improved quickly as the sample size increased. This may be because the correlation is a more complicated function of **n** than the mean, covariance, or standard deviation and therefore requires a larger sample size to stabilize. For the Fisher *Z*-transformation, SEs were not computed. Its CIs showed better coverage at 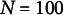 and coverage comparable to the proposed CIs at larger sample sizes.

#### 4.2.5 Split-half reliability coefficient and lambda coefficients

For all these coefficients, bias was small (Table 7). Similar to the sample correlation (Table 6), 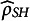 showed slight undercoverage for 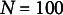 (Table 7), a pattern that is also shown in the supplementary material. Within the lambda series, 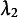 displayed slight undercoverage at 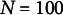 , while coverage was satisfactory for larger samples. In the supplementary material, the slight overcoverage for λ_1_ and λ_3_ in Table 7 is likely due to Monte Carlo error, as it did not appear under other conditions. By contrast, the CI for 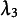 proposed by Feldt et al. (1987) exhibited systematic overcoverage (Table 7, last row).

**Table 6:** Bias of 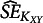 and coverage of the corresponding 95% Wald 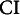 as estimated by the proposed method (upper panel), and the coverage of the CI estimated using the Fisher Z-transformation (lower panel)

Coef.	Par.	Scaled bias			Coverage		
		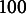	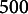	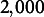	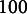	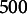	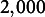
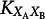	0.106	**−31.8%**	0.1%	−1.6%	**80.0%**	94.8%	95.0%
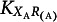	0.173	**−10.1%**	0.6%	1.2%	**79.7%**	94.5%	95.7%
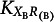	0.331	−1.6%	−0.4%	1.1%	94.0%	95.9%	94.5%
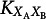	0.106				95.7%	95.2%	94.7%
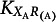	0.173				**96.3%**	95.7%	95.2%
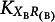	0.331				95.4%	95.0%	**96.0%**

*Note*: Coef. = coefficient, Par. = parameter value rounded to three decimals. Values 100, 500, and 2,000 in columns represent sample sizes. Item A is a low-mean dichotomous item, and item B is a high-variance dichotomous item (for details, see text). 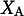 and 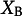 denote the scores on items A and B, respectively, and 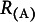 ad 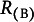 denote the rest scores of item A and B, respectively. Coverage percentages outside the 95% Agresti–Coull CI [93.9%, 95.8%] are shown in boldface. Scaled bias larger than 10% is shown in boldface.

**Table 7:** Bias of SEs of reliability estimates 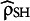 , 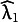 , 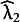 , and 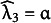 , and the coverage of the corresponding 95% Wald 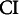

Coef.	Par.	Scaled bias			Coverage		
		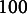	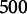	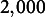	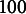	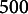	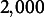
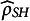	0.589	−3.9%	−0.7%	0.1%	**93.2%**	94.5%	94.8%
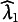	0.515	−4.3%	−0.3%	1.5%	93.8%	95.0%	**96.0%**
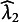	0.582	−3.7%	−0.3%	1.5%	**91.5%**	94.5%	95.1%
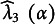	0.571	−4.4	−0.3	1.5%	93.8%	95.0%	**96.2%**
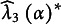	0.571	–	–	–	**96.0%**	**96.3%**	**97.0%**

*Note*: Coef. = coefficient, Par. = parameter value rounded to three decimals. Values 100, 500, and 2,000 in columns represent sample sizes. Coverage percentages outside the 95% Agresti–Coull CI [93.9%, 95.8%] are shown in boldface. Scaled bias larger than 10% is shown in boldface. * = CI as proposed by Feldt et al.(1987).

## 5 Discussion

This study provided analytic estimates of SEs and corresponding Wald CIs for 10 coefficients that are often used in the output of reliability analysis: mean, covariance, variance, standard deviation, product-moment correlation, reliability estimates 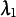 , 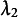 , and 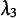 (better known as Cronbach’s alpha), and the split-half reliability coefficient. The SEs were derived using a two-step procedure: the scaled bias of the SEs and the coverage of the corresponding Wald CIs were further investigated in a simulation study and, when available, compared to other formula-based estimates of SEs and CIs. The derived SEs were implemented in the freely available software package JASP (as of version 19), and all coefficients and SEs are freely available as R functions on the Open Science Framework repository (Van der Ark, 2025).

In general, the scaled bias of the derived SEs was satisfactory for larger samples and coefficients whose values were not too close to the boundary of the parameter space. For more intricate coefficients (e.g., 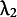 ), larger sample sizes were required than for a simple one (e.g., mean). For the covariance, variance, and standard deviation, other analytic SEs were available, which generally had a higher scaled bias than large-sample SEs. For the mean, it is well known that the conventional SE, based on the unbiased variance estimate, is unbiased, whereas the large-sample SE, based on the maximum-likelihood variance estimator, is not. Hence, for the mean, the conventional SE should always be preferred.

Similarly, the coverage of the Wald CIs, based on large-sample SEs, was satisfactory for larger samples and coefficients whose values were not too close to the boundary of the parameter space. For the correlation and 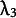 (Cronbach’s alpha), an asymmetric analytic CI was available. For correlation coefficients, for small samples ( 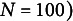 , Fisher-*Z* transformation resulted in a better coverage than Wald-based CIs. However, for 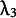 , the transformation of Feldt et al. (1987) did not result in better coverage than Wald CI.

If a coefficient lies close to a parameter boundary, sampling distribution tends to be skewed rather than normal, so Wald-based CIs may fail to be range-preserving and may exhibit undercoverage. For example, for 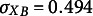 , the coverage of the CI was too low in the simulation study, which can be because the value of 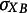 is close to its bound 0.5. In behavioral sciences, two types of bounds may be distinguished. First, coefficients may have theoretical bounds (e.g., 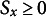 , 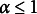 , 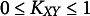 ). Second, coefficients may be bounded due to the data format. For example, for a single dichotomous item, the mean is bounded by 0 and 1, the variance by 0 and 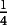 , and the standard deviation by 0 and 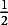 . For two dichotomous items, the covariance is bounded by 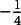 and 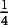 . These bounds also restrict the mean, variance, standard deviation, and covariance of composite scores, such as the sum score or rest score. In addition, these bounds also restrict coefficients that are functions of the coefficients, such as Cronbach’s alpha ( 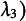 , which is a function of the variance–covariance matrix.

For Cronbach’s alpha, a series of papers has investigated its asymptotic distribution. Van Zyl et al. (2000) derived an asymptotic normal distribution without restrictions on the covariance structure, and this result was extended to nonnormal distributions by Ogasawara (2006) and Maydeu-Olivares et al. (2007). Some comparisons have been carried out; for example, Kuijpers et al. (2013b, Table 1) examined tests of the null hypothesis that Cronbach’s alpha equals a constant ccc and found that type I error rates were slightly better for the marginal modeling approach (from which the two-step procedure in this paper is derived) than for the method of Maydeu-Olivares et al. Nonetheless, a thorough comparison of different methods remains an important topic for future research.

The two-step procedure that was used to derive large-sample SEs assumes a multinomial distribution. On the one hand, by only assuming a multinomial distribution, the procedure is flexible with respect to the shape of the data distribution and can be applied to most coefficients used in social and behavioral research. In addition, the method can also be used to compute the asymptotic variance–covariance matrix between the coefficients. For example, in Appendix F, the asymptotic covariances of the sample variances and covariances were derived. On the other hand, as multinomial distribution is a discrete distribution, the procedure requires discrete scores (item scores, sum scores, and rest scores). Although the scores need not be integer-valued, for data in which virtually all respondents have distinct scores (e.g., time to complete a task measured in milliseconds), the procedure becomes time-consuming because each observed score is considered a separate category. This is particularly true when the asymptotic covariance matrix of the sample covariance matrix of the scores is required. In such cases, it is advisable to use alternative methods for continuous data. For example, for continuous data, the software package JASP computes SEs of the lambda coefficients under the assumption of an inverse Wishart distribution.

The study focused on analytic SEs, but SEs and CIs based on resampling, such as the nonparametric bootstrap (Efron & Tibshirani, 1986), or simulation, such as Mandel’s (2013) simulation algorithm that replaces the delta method. Although these methods may also provide good or even better results with respect to bias and coverage, analytic SEs have several advantages over SEs and CIs based on resampling or simulation, which make them useful in their own right. First, they are efficient in the sense that, once derived, they are very fast to compute and do not require thousands of resamples. Second, they are deterministic; they give the same result every time without Monte Carlo error. Third, they are transparent; they clarify the mathematical relationship between the SE and the factors affecting the SE. Moreover, analytic SEs facilitate sample-size planning, since closed-form SEs can often be inverted to yield explicit formulas for the required sample size, unlike bootstrap-based Ses, which require computationally intensive simulations. A well-known case is the mean, where 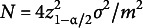 . Once these formulas for the required sample size have been derived, this advantage extends to other coefficients, such as reliability estimates or standard deviations.

## Supporting information

Van Der Ark supplementary material Van Der Ark supplementary material

## Footnotes

1
On August 30, 2025, Google Scholar reported 72,446 citations.

## Supplementary material

R code and supplementary materials are available on OSF (https://osf.io/y3bae).

## Appendix A: The estimated standard error of the sample mean

Let  be a  vector containing the unique observed scores of  (cf. Table 2, left panel). The sample mean can be written in the generalized exp-log notation as

(A1)

where  is a  matrix and  is a  row vector. Let  ; then, following Equations 2 and 3,

(A2)

and

(A3)

The Jacobian  can be derived from  and  . Let  . Following Equation 4,

(A4)

and following Equation 5,

(A5)

Because the sample mean is a scalar,  reduces to  . Furthermore, because  is homogeneous of order 0,  is given by Equation 12; that is,

(A6)

where  is the maximum-likelihood estimator of the variance, and the estimated standard error equals

Note that if  ,  (Equation A2) exists only as a complex number. This does not affect the theoretical derivation of the standard errors. The expression  (i.e., Equations A2 and A3 combined) is used only to divide  by  , and neither expression contains an imaginary part. The logarithm is not used in the derivation of the Jacobian. This reasoning applies to all derivations in this article.^2^ If  ,  does not exist, but replacing 0 with a small positive value (e.g.,  ) gave good results.

## Appendix B: The estimated standard error of the sample covariance

The unbiased covariance estimator equals  Let  be the  matrix containing the  possible response patterns with column vectors  and  , and let  denote the Hadamar (i.e., elementwise) product. Then,  can be written in the generalized exp-log notation as

(B1)

where  is a  matrix,  is a  matrix,  is a  matrix and  is a  row vector. With  ,  and cross-product  , following Equations 2 and 3,

(B2)

(B3)

(B4)

and

(B5)

Jacobian  can be derived from  , and  . Let  . Following Equations 4 and 5,

(B6)

(B7)

(B8)

and, the Jacobian equals

(B9)

Because the sample covariance is a scalar,  reduces to  , and because  is not homogeneous of order 0, Equation 14 should be used to compute  . The  th element of  (Equation B9) equals  . Let  be Kronecker delta; that is,  if  , and  otherwise. The estimated variance of  equals

(B10)

Rather than in terms of frequencies for response patterns  and  , Equation B10 can be rewritten for individual observations  and  (hence, the frequencies  and  in Equation B10 reduce to 1), resulting in

(B11)

With  (see Equation 15), the estimated standard error equals

(B12)

The expression  in Equation 16 equals

(B13)

For large  ,  , and replacing  by  in the last term of Equation B13 has neglible effect on the outcome. When doing so, that term becomes 0. As a result, for large

(B14)

## Appendix C: The estimated standard error of the sample standard deviation

The estimator of the standard deviation equals the square root of the unbiased estimator of the variance; that is,  which can be written in generalized exp-log notation: let  and Let  , both be  scalars. With  , and following Equations 2 and 3,

(C1)

and

(C2)

The Jacobian  can be derived from  With  , and following Equations 4 and 5,

(C3)

and

(C4)

Following Equation 11 and using the result from Equation C4, the estimated variance of  equals

(C5)

Then,  equals the square root of Equation C 5; that is,

(C6)

## Appendix D: The estimated standard error of the sample product-moment correlation

The sample product-moment correlation coefficient equals  Let  be the  matrix containing the  possible response patterns with column vectors  and  , and let  denote the Hadamar product. Then,  can be written in the generalized exp-log notation as

(D1)

where  is a  matrix,  is a  matrix,  is a  , and  is a  row vector. With  ,

(D2)

(D3)

(D4)

and

(D5)

The Jacobian  can be derived from  , and  . Let  . Following Equations 4 and 5,

(D6)

(D7)

(D8)

and, the Jacobian equals

(D9)

Because  is homogeneous of order 0,  is given by Equation 12; that is,

(D10)

Writing Equation D10 at the respondent level (see Appendix B) means that the  and yields

(D11)

The estimated SE is the square root of Equation D11; that is,

(D12)

## Appendix E: Split-half reliability coefficient

Let the test items be split into two sets  and  , and let  be the correlation between the sum scores on  and  . The sample value of the split-half reliability coefficient is  2  . The estimated variance of a correlation coefficient was derived in Appendix D (Equation D11); that is,

(E1)

Using  as a basis, the split-half reliability coefficient can be written in generalized exp-log notation: let  be a  vector, let  be a  matrix, let  be a  matrix, and let  be a 1  row vector. With  , and following Equations 2 and 3,

(E2)

(E3)

(E4)

and

(E5)

The Jacobian  can be derived from  and  . With  and following Equations 4 and 5,

, (E6)

(E7)

(E8)

and

(E9)

Following Equation 9, the estimated variance of  equals

(E10)

Then,  equals the square root of Equation E8; that is,

(E11)

## Appendix F: The estimated variance–covariance matrix of the sample variance–covariance matrix

variables—denoted by  , where  has  observed scores—result in  response patterns. Let  , and let  be the  vector of all univariate and bivariate frequencies, where univariate frequencies are given twice. The latter allows treating variances and covariances the same, which is convenient for the derivation. For example, if

(F1)

then vectors containing the univariate frequencies given twice are  ,  , and  , and the vectors providing the bivariate frequencies are  ,  , etc. Then,

(F2)

Let  be the  design matrix so that  . The first  rows of  correspond to  ; the next  rows to  , the next  rows to  , and so on. Element (  ,  ) in  equals 1 if the  th response pattern contributes to the  th univariate or bivariate frequency, and 0 otherwise. For example, for the vector of observed frequencies in Equation F1, the first and the second row of  equal  , which pertains to observed univariate frequency  The asymptotic covariance matrix of the sample covariance matrix is derived using the univariate and bivariate frequencies in  , whereas for the coefficients in Appendices A through E, vector  was used. Using vector  is useful because both the sample covariance (Equation B5) and its Jacobian (Equation B9) require only the bivariate frequencies as displayed in Equation F2.

Let  be the  sample variance covariance matrix variables  , let  be a  column vector obtained by stacking the column vectors of  on top of one another, let  denote the direct sum, and let  denote the Kronecker product; then,  (Equation B5) can be generalized to a vector function for the vectorized sample variance–covariance matrix by

(F3)

where  is a  matrix,  is a  matrix,  is a  matrix, and  is a  matrix. Note that  ,  are block-diagonal matrices that are generalizations of  ,  in Appendix B. Straightforward generalizations of Equations B2, B3, B4, and B5 yield

The Jacobian  can be derived as follows. Let  ,  ,  ,   , and  . Following Equations 4 and 5,

(F4)

(F5)

(F6)

and

(F7)

Finally, because the elements are not homogeneous of order 0, the estimated asymptotic variance covariance matrix of the vectorized sample variance–covariance matrix is given by Equation 11; that is,

(F8)

The diagonal elements of  are the estimated asymptotic variances of the sample variances and sample covariances that were derived in Appendix B. The off-diagonal elements are the estimated asymptotic covariances of the sample variances and covariances. Due to the irregular nature in matrix  , Equation F8 could not be simplified. The elements of  are referred to as  , where  refers to an estimated variance of a sample variance,  and  refer to an estimated variance of a sample covariance,  and  refer to an estimated covariance of sample variances,  or  refer to an estimated covariance of a sample variance and a sample covariance, and in all other cases to an estimated covariance of sample covariances.

## Appendix G: Guttman’s lambda 1

The sample value of Guttman’s lambda 1 equals  , where  denotes the variance of item  and  denotes the sum-score variance. The generalized exp-log notation of  uses  (see Appendix F) as a starting point. Let  be the number of items; then,  be a  matrix, where the elements of  equal 1 if they correspond to a variance (using the order of variances and covariances in  ) and equal 0 otherwise. Furthermore, let  be a  matrix and let  be a  row vector. With  and following Equations 2 and 3, it follows that

(G1)

(G2)

and

(G3)

Jacobian  can be derived from  and  . Let  . Following Equations 2 and 3,

(G4)

(G5)

and, the Jacobian equals

(G6)

Following Equation 9,

(G7)

is a weighted sum of the elements of  : elements that pertain to variances have weight  , all other elements have weight  . The estimated SE is

(G8)

Let  denote the covariance of sample covariances  and  (see Appendix F), and let  if  or  (i.e., at least one sample variance is involved); then, Equation G8 can be written as

(G9)

## Appendix H: Guttman’s lambda 2

Let  denote the number of items, let  be the sample inter-item variances (  ) and covariances (  ), let  denote the sum-score variance, let  denote the sum of the  item variances, let  denote the sum of all covariances, let  denote the sum of all squared covariances, and let  . The sample value of Guttman’s lambda 2 then equals

(H1)

where the right-hand side of Equation H1 is a convenient way to write  in the generalized exp-log notation. Let  denote the vectorized sample inter-item variance covariance matrix (see Appendix F), and let  be a  matrix—where  is a diagonal matrix with  on its diagonal and the elements of the  vector  equal 1 if they correspond to a variance in  and equal 0 if they correspond to a covariance in  . Furthermore, let  be a  matrix, where  denotes a diagonal matrix with the value 2 on all diagonal elements, let  be a  matrix, let  be a  matrix, let  be a  matrix, and let  be a  row vector. With  , and following Equations 2 and 3, it follows that

(H2)

where  the inter-item variance covariance matrix with all variances set to zero;

(H3)

where  a  matrix with the squared inter-item covariances on the off-diagonal elements and 0 on-diagonal elements;

(H4)

(H5)

(H6)

and

(H7)

The Jacobian  can be derived from  . Let  . Following Equations 4 and 5,

(H8)

where  is a diagonal matrix, with the elements corresponding to  (  ) equal to  , and all other elements equal to 0;

(H9)

(H10)

(H11)

(H12)

and

(H13)

Let  denote the estimated variance covariance matrix of  , with elements  (see Appendix F); then, following Equation 9,

(H14)

Let  be the element of  that pertains to (co)variance  (  ). It can be shown that the scalar notation of the last term of Equation H14 equals

(H15)

To simplify notation, let  ,  , and  . Then, Equation H15 reduces to

(H16)

Then, Equation H14 in scalar notation becomes

(H17)

So, the SE of  equals

(H18)
