# Supplementary material for: Standard Errors for Reliability Coefficients
Source: Psychometrika. 2025 Sep 30;90(5):1679–704. doi: 10.1017/psy.2025.10050 (PMC12805205; doi:10.1017/psy.2025.10050)
Supplement: Van Der Ark supplementary material [file S0033312325100501sup001.docx]

**Mean**

Item A

| I | M | D | P | Bias | | | Coverage | | |
| --- | --- | --- | --- | --- | --- | --- | --- | --- | --- |
|  |  |  |  | *N*=100 | *N*=500 | *N*=2000 | *N*=100 | *N*=500 | *N*=2000 |
| 10 | 2PL | 1 | .910 | 0.7 | 2.1 | -0.7 | 94.5 | 96.2 | 95.0 |
|  |  | 2 | .910 | -1.3 | 0.2 | 0.2 | 94.1 | 94.5 | 94.8 |
|  | AM | 1 | .827 | -1.5 | -0.2 | 1.6 | **91.8** | 94.8 | 94.7 |
|  |  | 2 | .828 | -0.3 | -0.1 | -1.6 | **92.4** | 95.4 | 95.1 |
| 30 | 2PL | 1 | .910 | 0.4 | 2.6 | -2.2 | 94.7 | 95.2 | 94.9 |
|  |  | 2 | .910 | 1.3 | 2.3 | 1.2 | 95.1 | 94.9 | 95.3 |
|  | AM | 1 | .827 | -1.6 | 1.4 | 1.7 | **93.5** | 95.0 | 94.8 |
|  |  | 2 | .828 | -2.5 | 1.2 | -0.5 | 94.5 | 95.2 | 94.1 |

Item B

| I | M | D | P | Bias | | | Coverage | | |
| --- | --- | --- | --- | --- | --- | --- | --- | --- | --- |
|  |  |  |  | *N*=100 | *N*=500 | *N*=2000 | *N*=100 | *N*=500 | *N*=2000 |
| 10 | 2PL | 1 | .575 | 1.2 | 1.1 | 1.5 | 94.9 | 95.2 | 95.2 |
|  |  | 2 | .575 | 0.9 | 2.8 | -3.7 | 95.0 | 95.5 | 93.9 |
|  | AM | 1 | .557 | 0.1 | 1.0 | -0.6 | 95.4 | 95.1 | 94.9 |
|  |  | 2 | .557 | -1.0 | -0.9 | -2.2 | 95.2 | 94.2 | 94.8 |
| 30 | 2PL | 1 | .575 | -0.6 | 3.6 | -0.9 | 94.5 | **96.0** | 94.6 |
|  |  | 2 | .575 | -2.8 | 0.4 | -0.4 | 94.1 | 95.0 | 94.8 |
|  | AM | 1 | .557 | 0.0 | -0.2 | 0.3 | 95.5 | 95.0 | 94.4 |
|  |  | 2 | .557 | -1.6 | 0.8 | 1.8 | 94.5 | 94.8 | 95.1 |

Total score

| I | M | D | P | Bias | | | Coverage | | |
| --- | --- | --- | --- | --- | --- | --- | --- | --- | --- |
|  |  |  |  | *N*=100 | *N*=500 | *N*=2000 | *N*=100 | *N*=500 | *N*=2000 |
| 10 | 2PL | 1 | 4.950 | -1.3 | 0.9 | 3.4 | 94.3 | 95.2 | 95.6 |
|  |  | 2 | 4.951 | -1.0 | 2.0 | -0.5 | 94.5 | 95.4 | 94.3 |
|  | AM | 1 | 4.915 | -1.3 | 2.1 | 1.0 | 94.7 | **95.9** | 95.2 |
|  |  | 2 | 4.918 | -2.0 | -0.6 | -0.7 | 94.7 | 95.0 | 95.0 |
| 30 | 2PL | 1 | 14.85 | -2.7 | -0.9 | 1.6 | 94.0 | 94.8 | **96.2** |
|  |  | 2 | 14.85 | -1.5 | -1.1 | 2.5 | 94.6 | **96.4** | 95.4 |
|  | AM | 1 | 14.75 | -1.6 | 0.3 | 1.3 | 95.0 | 94.8 | 95.1 |
|  |  | 2 | 14.76 | -2.6 | -0.4 | -0.1 | 94.5 | 94.8 | 95.1 |

*Note*. I = number of items, M = model, D = Number of dimensions, P = Parameter value, N = Sample size, 2PL = Two-parameter logistic model. AM = Acceleration model. Bold-faced percentages of scaled bias are greater than 10%. Bold faced coverages percentage lie outside the Agresti-Coull 95% confidence interval [93.95%, 95,88%]. Item parameters that generated the coefficients are the (partially) same across conditions, so conditions within a table are not independent.

**Variance**

Item A

| I | M | D | P | Bias | | | Coverage | | |
| --- | --- | --- | --- | --- | --- | --- | --- | --- | --- |
|  |  |  |  | *N*=100 | *N*=500 | *N*=2000 | *N*=100 | *N*=500 | *N*=2000 |
| 10 | 2PL | 1 | .082 | -0.0 | 1.8 | -0.8 | **93.6** | 95.7 | 95.0 |
|  |  | 2 | .082 | -1.7 | 0.1 | 0.1 | **93.1** | 94.5 | 94.8 |
|  | AM | 1 | .143 | -2.5 | -0.4 | 1.4 | **93.3** | **93.8** | 94.7 |
|  |  | 2 | .143 | -1.1 | -0.2 | -1.6 | 94.0 | 94.1 | 94.8 |
| 30 | 2PL | 1 | .082 | -0.3 | 2.4 | -2.3 | 94.0 | 94.9 | 94.7 |
|  |  | 2 | .082 | 0.8 | 2.2 | 1.1 | 94.0 | 94.9 | 95.3 |
|  | AM | 1 | .143 | -2.3 | 1.2 | 1.6 | **93.5** | 94.5 | 95.0 |
|  |  | 2 | .143 | -3.2 | 1.3 | -0.6 | **93.0** | 94.8 | 94.1 |

Item B

| I | M | D | P | Bias | | | Coverage | | |
| --- | --- | --- | --- | --- | --- | --- | --- | --- | --- |
|  |  |  |  | *N*=100 | *N*=500 | *N*=2000 | *N*=100 | *N*=500 | *N*=2000 |
| 10 | 2PL | 1 | .244 | -1.9^1^ | 0.5 | 1.2 | **84.1**^1^ | **89.3** | **93.0** |
|  |  | 2 | .244 | -1.22^2^ | 0.2 | -4.0 | **82.9**^2^ | **89.6** | **92.8** |
|  | AM | 1 | .247 | -5.5^3^ | -3.6^4^ | -1.4 | **82.0**^3^ | **87.7**^4^ | **92.3** |
|  |  | 2 | .247 | -4.0^5^ | -4.6^6^ | -3.2 | **82.2**^5^ | **87.9**^6^ | **92.4** |
| 30 | 2PL | 1 | .244 | -4.9^7^ | 2.1 | -1.7 | **82.7**^7^ | **90.0** | **93.5** |
|  |  | 2 | .244 | -7.9^8^ | -2.6 | -1.2 | **81.3**^8^ | **90.0** | **93.8** |
|  | AM | 1 | .247 | 0.6^9^ | -3.6^10^ | -1.3 | **82.9**^9^ | **88.2**^10^ | **92.2** |
|  |  | 2 | .247 | -1.3^11^ | -2.5^12^ | 0.4 | **81.7**^11^ | **87.9**^12^ | **93.7** |

Total score

| I | M | D | P | Bias | | | Coverage | | |
| --- | --- | --- | --- | --- | --- | --- | --- | --- | --- |
|  |  |  |  | *N*=100 | *N*=500 | *N*=2000 | *N*=100 | *N*=500 | *N*=2000 |
| 10 | 2PL | 1 | 3.247 | -1.4 | 0.5 | 0.7 | **93.2** | 94.8 | 95.4 |
|  |  | 2 | 2.273 | -0.8 | 1.9 | 2.0 | **93.2** | 95.2 | 95.2 |
|  | AM | 1 | 2.999 | -0.8 | 0.5 | 0.5 | **94.2** | 95.3 | 95.3 |
|  |  | 2 | 2.665 | -1.8 | 2.0 | 0.3 | **93.2** | 95.2 | 95.8 |
| 30 | 2PL | 1 | 21.22 | -5.1 | -0.5 | 1.1 | **93.2** | 95.2 | 95.5 |
|  |  | 2 | 17.49 | -2.7 | -0.8 | 0.6 | **92.7** | 94.7 | 94.8 |
|  | AM | 1 | 16.45 | -2.6 | -1.5 | 0.8 | **93.5** | 94.0 | 95.1 |
|  |  | 2 | 14.05 | -0.1 | -0.0 | 1.4 | 94.2 | 94.5 | 95.7 |

*Note*. I = number of items, M = model, D = Number of dimensions, P = Parameter value, N = Sample size, 2PL = Two-parameter logistic model. AM = Acceleration model. Bold-faced percentages of scaled bias are greater than 10%. Bold faced coverages percentage lie outside the Agresti-Coull 95% confidence interval [93.95%, 95,88%] . Item parameters that generated the coefficients are the (partially) same across conditions, so conditions within a table are not independent. In some replications the SE of the variance of item B could not be computed because the sample variance equaled 0. The number of replications were: ^1^1949, ^2^1942, ^3^1921, ^4^1998, ^5^1931, ^6^1996, ^7^1945, ^8^1942, ^9^1933, ^10^1998, ^11^1930, ^12^1997.

**Standard deviation**

Item A

| I | M | D | P | Bias | | | Coverage | | |
| --- | --- | --- | --- | --- | --- | --- | --- | --- | --- |
|  |  |  |  | *N*=100 | *N*=500 | *N*=2000 | *N*=100 | *N*=500 | *N*=2000 |
| 10 | 2PL | 1 | .286 | -2.0 | 1.4 | -1.0 | 94.8 | **96.0** | 95.0 |
|  |  | 2 | .286 | -2.7 | -0.2 | -0.2 | 94.3 | 95.5 | 95.0 |
|  | AM | 1 | .378 | -3.3 | -0.5 | 1.3 | **93.0** | 94.7 | 95.2 |
|  |  | 2 | .378 | -1.9 | -0.3 | -1.5 | **93.4** | 95.0 | 94.9 |
| 30 | 2PL | 1 | .286 | -1.6 | 1.9 | -2.4 | 95.2 | 95.4 | 94.6 |
|  |  | 2 | .286 | -0.0 | 2.2 | 0.9 | 95.5 | 95.5 | 95.3 |
|  | AM | 1 | .378 | -2.8 | 1.1 | 1.5 | **93.0** | 95.0 | 95.3 |
|  |  | 2 | .378 | -3.8 | 1.3 | -0.7 | **92.7** | 95.2 | 94.1 |

Item B

| I | M | D | P | Bias | | | Coverage | | |
| --- | --- | --- | --- | --- | --- | --- | --- | --- | --- |
|  |  |  |  | *N*=100 | *N*=500 | *N*=2000 | *N*=100 | *N*=500 | *N*=2000 |
| 10 | 2PL | 1 | .494 | -2.4^1^ | 0.5 | 1.1 | **84.1**^1^ | **89.5** | **93.0** |
|  |  | 2 | .494 | -1.7^2^ | 0.1 | -4.0 | **82.9**^2^ | **89.7** | **92.8** |
|  | AM | 1 | .497 | -6.0^3^ | -3.7^4^ | -1.4 | **82.0**^3^ | **87.7**^4^ | **92.3** |
|  |  | 2 | .497 | -4.6^5^ | -4.7^6^ | -3.3 | **82.2**^5^ | **87.9**^6^ | **92.5** |
| 30 | 2PL | 1 | .494 | -5.2^7^ | 2.1 | -1.7 | **82.9**^7^ | **90.2** | **93.5** |
|  |  | 2 | .494 | -8.4^8^ | -2.8 | -1.2 | **81.4**^8^ | **90.1** | **93.8** |
|  | AM | 1 | .497 | -2.9^9^ | -1.5^10^ | 0.8 | **82.9**^9^ | **88.2**^10^ | **92.2** |
|  |  | 2 | .497 | -1.7^11^ | -2.6^12^ | 0.3 | **81.8**^11^ | **87.9**^12^ | **93.7** |

Total score

| I | M | D | P | Bias | | | Coverage | | |
| --- | --- | --- | --- | --- | --- | --- | --- | --- | --- |
|  |  |  |  | *N*=100 | *N*=500 | *N*=2000 | *N*=100 | *N*=500 | *N*=2000 |
| 10 | 2PL | 1 | 1.802 | -1.7 | 0.4 | 0.7 | **93.4** | 95.0 | 95.2 |
|  |  | 2 | 1.650 | -1.0 | 1.9 | 2.0 | 94.1 | 95.2 | 95.2 |
|  | AM | 1 | 1.732 | -0.9 | 0.5 | 0.5 | 94.3 | 95.2 | 95.5 |
|  |  | 2 | 2.665 | -1.8 | 2.0 | 0.3 | **93.2** | 95.2 | 95.8 |
| 30 | 2PL | 1 | 4.606 | -5.0 | -0.5 | 1.1 | **93.6** | 95.2 | 95.4 |
|  |  | 2 | 4.182 | -3.2 | -0.7 | 0.7 | **93.1** | 94.5 | 94.8 |
|  | AM | 1 | 4.056 | -2.9 | -1.5 | 0.8 | 94.0 | 94.0 | 95.3 |
|  |  | 2 | 3.749 | -0.3 | 0.0 | 1.4 | 94.6 | 95.0 | 95.3 |

*Note*. I = number of items, M = model, D = Number of dimensions, P = Parameter value, N = Sample size, 2PLM = Two-parameter logistic model. AM = Acceleration model. Bold-faced percentages of scaled bias are greater than 10%. Bold faced coverages percentage lie outside the Agresti-Coull 95% confidence interval [93.95%, 95,88%]. Item parameters that generated the coefficients are the (partially) same across conditions, so conditions within a table are not independent. In some replications the SE of the SD of item B could not be computed because the sample SD equaled 0. The number of replications were: ^1^1949, ^2^1942, ^3^1921, ^4^1998, ^5^1931, ^6^1996, ^7^1945, ^8^1942, ^9^1933, ^10^1998, ^11^1930, ^12^1997.

**Alternative standard deviation**

Item A

| I | M | D | P | Bias | | | Coverage | | |
| --- | --- | --- | --- | --- | --- | --- | --- | --- | --- |
|  |  |  |  | *N*=100 | *N*=500 | *N*=2000 | *N*=100 | *N*=500 | *N*=2000 |
| 10 | 2PL | 1 | .286 | **-52.0** | **50.3** | **-51.3** | **71.2** | **64.2** | **65.7** |
|  |  | 2 | .286 | **-52.7** | **-51.2** | **-50.1** | **68.9** | **63.3** | **67.6** |
|  | AM | 1 | .378 | **-21.6** | **-19.0** | **-17.4** | **87.5** | **87..9** | **89.3** |
|  |  | 2 | .378 | **-19.9** | **-18.4** | **-19.6** | **88.0** | **88.6** | **88.6** |
| 30 | 2PL | 1 | .286 | **-52.4** | **-50.1** | **-52.0** | **69.2** | **67.0** | **64.3** |
|  |  | 2 | .286 | **-51.2** | **-49.7** | **-50.3** | **70.4** | **66.4** | **66.1** |
|  | AM | 1 | .378 | **-21.0** | **-17.3** | **-17.2** | **87.4** | **88.9** | **90.3** |
|  |  | 2 | .378 | **-22.5** | **-17.7** | **-19.2** | **87.1** | **89.0** | **88.4** |

Item B

| I | M | D | P | Bias | | | Coverage | | |
| --- | --- | --- | --- | --- | --- | --- | --- | --- | --- |
|  |  |  |  | *N*=100 | *N*=500 | *N*=2000 | *N*=100 | *N*=500 | *N*=2000 |
| 10 | 2PL | 1 | .494 | **318** | **367** | **370** | **100** | **100** | **100** |
|  |  | 2 | .494 | **332** | **366** | **346** | **100** | **100** | **100** |
|  | AM | 1 | .497 | **395** | **494** | **507** | **100** | **100** | **100** |
|  |  | 2 | .497 | **408** | **483** | **494** | **100** | **100** | **100** |
| 30 | 2PL | 1 | .494 | **308** | **372** | **355** | **100** | **100** | **100** |
|  |  | 2 | .494 | **301** | **346** | **358** | **100** | **100** | **100** |
|  | AM | 1 | .497 | **438** | **490** | **509** | **100** | **100** | **100** |
|  |  | 2 | .497 | **438** | **490** | **509** | **100** | **100** | **100** |

Total score

| I | M | D | P | Bias | | | Coverage | | |
| --- | --- | --- | --- | --- | --- | --- | --- | --- | --- |
|  |  |  |  | *N*=100 | *N*=500 | *N*=2000 | *N*=100 | *N*=500 | *N*=2000 |
| 10 | 2PL | 1 | 1.802 | 8.4 | 9.6 | 9.8 | **95.9** | **96.7** | **96.9** |
|  |  | 2 | 1.650 | 7.1 | 8.9 | 8.6 | 95.1 | **96.2** | **96.5** |
|  | AM | 1 | 1.732 | 8.9 | 8.9 | 9.7 | **96.5** | **97.2** | **96.5** |
|  |  | 2 | 1.665 | 6.8 | 9.0 | 6.7 | **95.9** | **96.8** | **96.9** |
| 30 | 2PL | 1 | 4.606 | 5.7 | 9.1 | **10.7** | 95.5 | **97.4** | **96.9** |
|  |  | 2 | 4.182 | 5.6 | 6.8 | 7.9 | 95.5 | **96.3** | **96.2** |
|  | AM | 1 | 4.056 | 6.4 | 6.4 | 8.7 | **96.5** | 95.7 | **96.6** |
|  |  | 2 | 3.749 | 7.6 | 6.5 | 7.5 | **96.7** | **96.4** | **96.2** |

*Note*. I = number of items, M = model, D = Number of dimensions, P = Parameter value, N = Sample size, 2PLM = Two-parameter logistic model. AM = Acceleration model. Bold-faced percentages of scaled bias are greater than 10%. Bold faced coverages percentage lie outside the Agresti-Coull 95% confidence interval [93.95%, 95,88%]. Item parameters that generated the coefficients are the (partially) same across conditions, so conditions within a table are not independent.

**Covariance**

Item A and item B

| I | M | D | P | Bias | | | Coverage | | |
| --- | --- | --- | --- | --- | --- | --- | --- | --- | --- |
|  |  |  |  | *N*=100 | *N*=500 | *N*=2000 | *N*=100 | *N*=500 | *N*=2000 |
| 10 | 2PL | 1 | .015 | 0.1 | 2.8 | -1.4 | 95.2 | 95.7 | 95.0 |
|  |  | 2 | .015 | -2.8 | -0.8 | 0.6 | **93.8** | 95.0 | **96.0** |
|  | AM | 1 | .010 | -2.3 | 0.6 | 1.1 | 94.2 | 95.2 | 95.6 |
|  |  | 2 | .010 | 0.7 | 0.8 | -1.3 | 95.1 | 95.0 | 95.0 |
| 30 | 2PL | 1 | .015 | -5.6 | 1.6 | 1.7 | **92.2** | 95.1 | 95.5 |
|  |  | 2 | .015 | -1.8 | 1.9 | -0.9 | 94.0 | 95.5 | **95.9** |
|  | AM | 1 | .010 | -4.0 | 0.2 | 3.0 | **93.4** | 94.5 | 95.5 |
|  |  | 2 | .010 | -3.3 | 1.0 | 3.2 | 94.0 | 94.8 | 95.7 |

Item A and rest score

| I | M | D | P | Bias | | | Coverage | | |
| --- | --- | --- | --- | --- | --- | --- | --- | --- | --- |
|  |  |  |  | *N*=100 | *N*=500 | *N*=2000 | *N*=100 | *N*=500 | *N*=2000 |
| 10 | 2PL | 1 | .086 | 1.4 | 2.1 | 0.5 | **93.8** | 95.3 | 95.2 |
|  |  | 2 | .071 | -5.0 | -0.5 | 0.8 | **91.6** | 94.0 | 95.2 |
|  | AM | 1 | .056 | -2.8 | -0.3 | -0.8 | 94.2 | 94.9 | 94.7 |
|  |  | 2 | .046 | -1.5 | -0.4 | -1.7 | 94.2 | 94.5 | 95.0 |
| 30 | 2PL | 1 | .268 | -6.4 | 1.0 | -0.4 | **90.8** | 94.4 | 94.5 |
|  |  | 2 | .224 | -3.8 | -0.4 | 0.5 | **91.2** | 94.3 | 94.8 |
|  | AM | 1 | .173 | -2.7 | -2.3 | 2.2 | 94.4 | 94.2 | 95.8 |
|  |  | 2 | .497 | -1.7 | -2.6 | 0.3 | 81.8 | 87.9 | **93.7** |

Item B and rest score

| I | M | D | P | Bias | | | Coverage | | |
| --- | --- | --- | --- | --- | --- | --- | --- | --- | --- |
|  |  |  |  | *N*=100 | *N*=500 | *N*=2000 | *N*=100 | *N*=500 | *N*=2000 |
| 10 | 2PL | 1 | .258 | -1.0 | 0.1 | 1.4 | 94.2 | 94.7 | 95.5 |
|  |  | 2 | .208 | -0.6 | 0.1 | 1.0 | 94.5 | 95.3 | 95.2 |
|  | AM | 1 | .165 | -1.1 | -1.2 | -0.4 | 94.2 | 94.7 | 94.5 |
|  |  | 2 | .134 | -0.2 | -1.3 | 0.2 | 94.6 | 95.4 | 95.0 |
| 30 | 2PL | 1 | .877 | -3.7 | 2.4 | -1.6 | **93.4** | 95.3 | 94.6 |
|  |  | 2 | .727 | -1.3 | -3.0 | -0.7 | 94.2 | 94.5 | 94.7 |
|  | AM | 1 | .561 | -4.0 | 1.4 | -1.1 | 94.0 | 94.2 | 94.8 |
|  |  | 2 | .465 | -2.5 | 1.2 | -0.4 | 94.9 | 95.7 | 94.2 |

*Note*. I = number of items, M = model, D = Number of dimensions, P = Parameter value, N = Sample size, 2PLM = Two-parameter logistic model. AM = Acceleration model. Bold-faced percentages of scaled bias are greater than 10%. Bold faced coverages percentage lie outside the Agresti-Coull 95% confidence interval [93.95%, 95,88%]. Item parameters that generated the coefficients are the (partially) same across conditions, so conditions within a table are not independent.

**Alternative Covariance**

Item A and item B

| I | M | D | P | Bias | | | Coverage | | |
| --- | --- | --- | --- | --- | --- | --- | --- | --- | --- |
|  |  |  |  | *N*=100 | *N*=500 | *N*=2000 | *N*=100 | *N*=500 | *N*=2000 |
| 10 | 2PL | 1 | .015 | **12.4** | **14.4** | 9.5 | **97.8** | **97.9** | **96.6** |
|  |  | 2 | .015 | 9.8 | **10.6** | **11.9** | **97.1** | **97.1** | **97.6** |
|  | AM | 1 | .010 | 1.5 | 3.7 | 3.9 | 95.6 | **96.2** | **96.2** |
|  |  | 2 | .010 | 4.5 | 3.8 | 1.5 | **96.2** | 95.7 | 95.7 |
| 30 | 2PL | 1 | .015 | 6.6 | **13.2** | **13.0** | **96.0** | **97.5** | **97.5** |
|  |  | 2 | .015 | **10.8** | **13.3** | **10.0** | **97.2** | **97.4** | **97.4** |
|  | AM | 1 | .010 | -0.3 | 3.2 | 6.0 | 94.7 | 95.5 | **96.2** |
|  |  | 2 | .010 | 0.4 | 4.1 | 6.3 | 95.0 | 95.7 | **96.2** |

Item A and rest score

| I | M | D | P | Bias | | | Coverage | | |
| --- | --- | --- | --- | --- | --- | --- | --- | --- | --- |
|  |  |  |  | *N*=100 | *N*=500 | *N*=2000 | *N*=100 | *N*=500 | *N*=2000 |
| 10 | 2PL | 1 | .086 | **204** | **200** | **194** | **100** | **100** | **100** |
|  |  | 2 | .071 | **165** | **172** | **173** | **100** | **100** | **100** |
|  | AM | 1 | .056 | 125 | 126 | 125 | **100** | **100** | **100** |
|  |  | 2 | .046 | **115** | **115** | **111** | **100** | **100** | **100** |
| 30 | 2PL | 1 | .268 | **598** | **630** | **615** | **100** | **100** | **100** |
|  |  | 2 | .224 | **555** | **559** | **562** | **100** | **100** | **100** |
|  | AM | 1 | .173 | **411** | **404** | **426** | **100** | **100** | **100** |
|  |  | 2 | .497 | **371** | **382** | **375** | **100** | **100** | **100** |

Item B and rest score

| I | M | D | P | Bias | | | Coverage | | |
| --- | --- | --- | --- | --- | --- | --- | --- | --- | --- |
|  |  |  |  | *N*=100 | *N*=500 | *N*=2000 | *N*=100 | *N*=500 | *N*=2000 |
| 10 | 2PL | 1 | .258 | **84.6** | **85.7** | **88.0** | **100** | **99.9** | **100** |
|  |  | 2 | .208 | **69.5** | **69.9** | **71.4** | **99.9** | **99.9** | **99.9** |
|  | AM | 1 | .165 | **76.5** | **74.4** | **75.7** | **99.8** | **99.9** | **99.9** |
|  |  | 2 | .134 | **68.2** | **65.3** | **67.7** | **99.8** | **99.9** | **99.9** |
| 30 | 2PL | 1 | .877 | **373** | **401** | **381** | **100** | **100** | **100** |
|  |  | 2 | .727 | **334** | **324** | **333** | **100** | **100** | **100** |
|  | AM | 1 | .561 | **301** | **321** | **309** | **100** | **100** | **100** |
|  |  | 2 | .465 | **273** | **285** | **278** | **100** | **100** | **100** |

*Note*. I = number of items, M = model, D = Number of dimensions, P = Parameter value, N = Sample size, 2PLM = Two-parameter logistic model. AM = Acceleration model. Bold-faced percentages of scaled bias are greater than 10%. Bold faced coverages percentage lie outside the Agresti-Coull 95% confidence interval [93.95%, 95,88%]. Item parameters that generated the coefficients are the (partially) same across conditions, so conditions within a table are not independent.

**Correlation**

Item A and item B

| I | M | D | P | Bias | | | Coverage | | |
| --- | --- | --- | --- | --- | --- | --- | --- | --- | --- |
|  |  |  |  | *N*=100 | *N*=500 | *N*=2000 | *N*=100 | *N*=500 | *N*=2000 |
| 10 | 2PL | 1 | .106 | **-31.8** | 0.1 | -1.6 | **80.0** | 94.8 | 95.0 |
|  |  | 2 | .106 | **-34.9** | -2.5 | 0.7 | **79.7** | 94.5 | 95.7 |
|  | AM | 1 | .051 | **-63.2** | **-22.8** | -0.6 | **65.5** | **86.2** | 95.8 |
|  |  | 2 | .051 | -**60.1** | -**22.6** | -3.5 | **67.4** | **86.0** | 95.0 |
| 30 | 2PL | 1 | .107 | **-37.3** | -1.2 | 1.9 | **78.2** | 94.8 | 95.8 |
|  |  | 2 | .107 | **-35.0** | -0.3 | -1.6 | **79.6** | 95.2 | 95.1 |
|  | AM | 1 | .051 | **-62.4** | **-26.1** | 0.3 | **65.2** | **84.2** | 95.5 |
|  |  | 2 | .051 | **-60.0** | -24.4 | 1.4 | **66.3** | **84.5** | 95.6 |

Item A and rest score

| I | M | D | P | Bias | | | Coverage | | |
| --- | --- | --- | --- | --- | --- | --- | --- | --- | --- |
|  |  |  |  | *N*=100 | *N*=500 | *N*=2000 | *N*=100 | *N*=500 | *N*=2000 |
| 10 | 2PL | 1 | .173 | **-10.1** | 0.6 | 1.2 | **91.6** | 94.8 | 94.8 |
|  |  | 2 | .157 | -**19.1** | -0.8 | -0.2 | **88.8** | 94.3 | 95.0 |
|  | AM | 1 | .089 | **-40.9** | -5.7 | -0.8 | **76.3** | 94.2 | 94.7 |
|  |  | 2 | .079 | **-44.6** | -9.2 | -2.1 | **74.9** | **92.8** | 95.0 |
| 30 | 2PL | 1 | .207 | -**15.0** | -0.9 | -0.1 | **90.5** | 95.0 | 94.3 |
|  |  | 2 | .191 | **-14.3** | -2.0 | -0.9 | **90.5** | 94.8 | 95.2 |
|  | AM | 1 | .114 | **-30.6** | -4.3 | 2.2 | **82.5** | 94.0 | 95.6 |
|  |  | 2 | .104 | -**34.6** | -1.1 | -0.9 | **80.6** | 95.9 | 94.5 |

Item B and rest score

| I | M | D | P | Bias | | | Coverage | | |
| --- | --- | --- | --- | --- | --- | --- | --- | --- | --- |
|  |  |  |  | *N*=100 | *N*=500 | *N*=2000 | *N*=100 | *N*=500 | *N*=2000 |
| 10 | 2PL | 1 | .331 | -1.6 | -0.4 | 1.1 | 94.0 | 94.5 | 95.2 |
|  |  | 2 | .293 | -1.3 | 0.1 | 0.9 | **93.7** | 95.3 | 94.9 |
|  | AM | 1 | .214 | -5.7 | -1.4 | -0.2 | **93.2** | 94.1 | 94.6 |
|  |  | 2 | .183 | -7.1 | -1.8 | 0.0 | **93.4** | 95.0 | 95.0 |
| 30 | 2PL | 1 | .405 | -3.9 | 2.5 | -1.9 | **92.7** | 95.2 | 94.5 |
|  |  | 2 | .370 | -1.5 | -2.4 | -1.9 | **93.5** | 94.7 | 94.9 |
|  | AM | 1 | .291 | -5.0 | 0.6 | -1.2 | **93.5** | 95.0 | 94.4 |
|  |  | 2 | .261 | -3.7 | 1.5 | -1.1 | 94.0 | 95.6 | 93.8 |

*Note*. I = number of items, M = model, D = Number of dimensions, P = Parameter value, N = Sample size, 2PLM = Two-parameter logistic model. AM = Acceleration model. Bold-faced percentages of scaled bias are greater than 10%. Bold faced coverages percentage lie outside the Agresti-Coull 95% confidence interval [93.95%, 95,88%]. Item parameters that generated the coefficients are the (partially) same across conditions, so conditions within a table are not independent.

**Correlation Alternative**

Item A and item B

| I | M | D | P | Bias | | | Coverage | | |
| --- | --- | --- | --- | --- | --- | --- | --- | --- | --- |
|  |  |  |  | *N*=100 | *N*=500 | *N*=2000 | *N*=100 | *N*=500 | *N*=2000 |
| 10 | 2PL | 1 | .107 | -0.0 | 0.8 | -3.1 | 95.7 | 95.2 | 94.7 |
|  |  | 2 | .107 | -1.9 | -1.6 | -0.8 | 94.8 | 94.8 | 95.5 |
|  | AM | 1 | .051 | -1.8 | 0.0 | 0.0 | 94.5 | 95.5 | 95.7 |
|  |  | 2 | .051 | 0.9 | 0.4 | -2.0 | 95.5 | 95.0 | 94.8 |
| 30 | 2PL | 1 | .107 | -5.9 | -0.2 | 0.4 | 93.4 | 95.0 | 95.2 |
|  |  | 2 | .107 | -1.2 | 0.3 | -3.1 | 95.2 | 95.2 | 95.0 |
|  | AM | 1 | .051 | -3.2 | -0.3 | 2.2 | **93.8** | 94.6 | 95.3 |
|  |  | 2 | .051 | -2.6 | 0.6 | 2.5 | 94.0 | 94.8 | 95.4 |

Item A and rest score

| I | M | D | P | Bias | | | Coverage | | |
| --- | --- | --- | --- | --- | --- | --- | --- | --- | --- |
|  |  |  |  | *N*=100 | *N*=500 | *N*=2000 | *N*=100 | *N*=500 | *N*=2000 |
| 10 | 2PL | 1 | .173 | 5.2 | 3.3 | 3.1 | **96.3** | 95.7 | 95.2 |
|  |  | 2 | .157 | -0.7 | 1.3 | 1.1 | 95.2 | 95.2 | 95.3 |
|  | AM | 1 | .089 | -2.1 | -1.6 | -1.8 | 94.5 | 94.6 | 94.5 |
|  |  | 2 | .079 | -0.7 | -1.4 | -2.8 | 94.5 | 94.4 | 94.9 |
| 30 | 2PL | 1 | .207 | -2.8 | 0.2 | 0.4 | 94.5 | 94.8 | 94.4 |
|  |  | 2 | .191 | -1.8 | -1.7 | -1.3 | 94.2 | 95.0 | 95.2 |
|  | AM | 1 | .114 | -2.8 | -4.7 | -0.2 | 94.6 | **93.8** | 95.0 |
|  |  | 2 | .104 | -3.8 | -1.4 | -3.0 | 94.4 | 94.8 | 94.0 |

Item B and rest score

| I | M | D | P | Bias | | | Coverage | | |
| --- | --- | --- | --- | --- | --- | --- | --- | --- | --- |
|  |  |  |  | *N*=100 | *N*=500 | *N*=2000 | *N*=100 | *N*=500 | *N*=2000 |
| 10 | 2PL | 1 | .331 | -1.6 | -0.4 | 1.1 | 95.4 | 95.0 | **96.0** |
|  |  | 2 | .293 | 2.8 | 2.7 | 3.2 | 95.0 | **96.1** | 95.5 |
|  | AM | 1 | .214 | 0.6 | -0.4 | 0.6 | 94.7 | 95.0 | 94.8 |
|  |  | 2 | .183 | 2.2 | 0.9 | 0.8 | 95.2 | 95.3 | 95.0 |
| 30 | 2PL | 1 | .405 | 1.3 | 6.5 | 1.6 | 95.2 | **96.0** | 95.5 |
|  |  | 2 | .370 | 3.5 | 0.8 | 1.1 | 95.5 | 95.0 | 95.5 |
|  | AM | 1 | .291 | -1.7 | 2.0 | -0.0 | 94.8 | 95.5 | 94.8 |
|  |  | 2 | .261 | -0.7 | 2.8 | 0.0 | 95.5 | **96.1** | 94.0 |

*Note*. I = number of items, M = model, D = Number of dimensions, P = Parameter value, N = Sample size, 2PLM = Two-parameter logistic model. AM = Acceleration model. Bold-faced percentages of scaled bias are greater than 10%. Bold faced coverages percentage lie outside the Agresti-Coull 95% confidence interval [93.95%, 95,88%]. Item parameters that generated the coefficients are the (partially) same across conditions, so conditions within a table are not independent.

**Lambda 1**

| I | M | D | P | Bias | | | Coverage | | |
| --- | --- | --- | --- | --- | --- | --- | --- | --- | --- |
|  |  |  |  | *N*=100 | *N*=500 | *N*=2000 | *N*=100 | *N*=500 | *N*=2000 |
| 10 | 2PL | 1 | .515 | -4.3 | -0.3 | 1.5 | 93.8 | 95.0 | **96.2** |
|  |  | 2 | .477 | -2.8 | -1.8 | 1.4 | 94.4 | 95.0 | 95.5 |
|  | AM | 1 | .362 | -2.4 | 0.4 | -0.0 | 94.2 | 95.2 | 95.6 |
|  |  | 2 | .319 | -4.4 | 0.3 | 1.5 | 93.8 | 95.0 | **96.2** |
| 30 | 2PL | 1 | .777 | -6.3 | -0.1 | 1.5 | **93.4** | 94.8 | 95.8 |
|  |  | 2 | .756 | -6.4 | -1.5 | 0.7 | **93.3** | 94.2 | 95.0 |
|  | AM | 1 | .651 | -4.5 | -1.8 | 0.2 | 93.9 | 94.6 | 95.3 |
|  |  | 2 | .612 | -2.2 | 0.5 | 1.1 | 94.8 | 95.5 | 95.2 |

**Lambda 2**

| I | M | D | P | Bias | | | Coverage | | |
| --- | --- | --- | --- | --- | --- | --- | --- | --- | --- |
|  |  |  |  | *N*=100 | *N*=500 | *N*=2000 | *N*=100 | *N*=500 | *N*=2000 |
| 10 | 2PL | 1 | .582 | -3.7 | -0.3 | 1.5 | **91.5** | 94.5 | **96.0** |
|  |  | 2 | .544 | -1.8 | 1.8 | 1.6 | **92.8** | 94.5 | 95.1 |
|  | AM | 1 | .410 | -1.6 | 0.6 | -0.3 | **90.2** | 93.8 | 94.6 |
|  |  | 2 | .363 | -2.4 | 2.3 | 0.3 | **90.8** | 94.8 | 95.5 |
| 30 | 2PL | 1 | .809 | -5.7 | 0.1 | 1.5 | **91.5** | 94.5 | 95.6 |
|  |  | 2 | .789 | -5.7 | -1.4 | 0.8 | **91.6** | **93.5** | 94.8 |
|  | AM | 1 | .677 | -4.1 | -1.8 | 0.3 | **90.0** | **93.5** | 95.2 |
|  |  | 2 | .639 | -1.8 | 0.5 | 1.1 | **90.2** | 94.6 | 94.7 |

**Lambda 3**

| I | M | D | P | Bias | | | Coverage | | |
| --- | --- | --- | --- | --- | --- | --- | --- | --- | --- |
|  |  |  |  | *N*=100 | *N*=500 | *N*=2000 | *N*=100 | *N*=500 | *N*=2000 |
| 10 | 2PL | 1 | .571 | -4.4 | -0.3 | 1.5 | 93.8 | 95.0 | **96.2** |
|  |  | 2 | .531 | -2.8 | -1.8 | 1.4 | 94.4 | 95.0 | 95.5 |
|  | AM | 1 | .403 | -2.4 | 0.4 | -0.0 | 94.2 | 95.2 | 95.6 |
|  |  | 2 | .354 | -2.9 | 2.2 | 0.5 | 90.8 | 95.5 | **96.0** |
| 30 | 2PL | 1 | .804 | -6.3 | -0.1 | 1.5 | **93.4** | 94.8 | 95.8 |
|  |  | 2 | .782 | -6.5 | -1.5 | 0.7 | **93.3** | 94.2 | 95.0 |
|  | AM | 1 | .674 | -4.5 | -1.9 | 0.3 | 93.9 | 94.6 | 95.3 |
|  |  | 2 | .633 | -2.2 | 0.5 | 1.1 | 94.8 | 95.5 | 95.2 |

*Note*: I = number of items, M = model, D = Number of dimensions, P = Parameter value, N = Sample size, 2PLM = Two-parameter logistic model. AM = Acceleration model. Bold-faced percentages of scaled bias are greater than 10%. Bold faced coverages percentage lie outside the Agresti-Coull 95% confidence interval [93.95%, 95,88%]. Item parameters that generated the coefficients are the (partially) same across conditions, so conditions within a table are not independent.

**Lambda 3 alternative**

| I | M | D | P | Bias | | | Coverage | | |
| --- | --- | --- | --- | --- | --- | --- | --- | --- | --- |
|  |  |  |  | *N*=100 | *N*=500 | *N*=2000 | *N*=100 | *N*=500 | *N*=2000 |
| 10 | 2PL | 1 | .571 |  |  |  | **96.0** | **96.3** | **97.0** |
|  |  | 2 | .531 |  |  |  | **96.6** | **96.9** | **96.9** |
|  | AM | 1 | .403 |  |  |  | 95.8 | **95.9** | 95.8 |
|  |  | 2 | .354 |  |  |  | **96.0** | **96.4** | **96.5** |
| 30 | 2PL | 1 | .804 |  |  |  | 95.5 | **96.8** | **96.8** |
|  |  | 2 | .782 |  |  |  | **96.6** | **96.4** | **96.1** |
|  | AM | 1 | .674 |  |  |  | **96.2** | 95.3 | **96.1** |
|  |  | 2 | .633 |  |  |  | 95.8 | **96.2** | **95.9** |

**Split-half correlation coefficient**

| I | M | D | P | Bias | | | Coverage | | |
| --- | --- | --- | --- | --- | --- | --- | --- | --- | --- |
|  |  |  |  | *N*=100 | *N*=500 | *N*=2000 | *N*=100 | *N*=500 | *N*=2000 |
| 10 | 2PL | 1 | .589 | -3.9 | -0.7 | 0.1 | **93.2** | 94.5 | 94.8 |
|  |  | 2 | .472 | -5.2 | 0.2 | -1.6 | **92.9** | 95.0 | 94.4 |
|  | AM | 1 | .414 | -5.6 | -0.6 | 0.0 | **92.5** | 95.0 | 95.5 |
|  |  | 2 | .314 | -7.8 | 1.3 | 3.8 | **91.2** | 94.9 | 95.7 |
| 30 | 2PL | 1 | .811 | -3.5 | -0.9 | -1.1 | **93.0** | 94.7 | 95.0 |
|  |  | 2 | .661 | -3.9 | -0.5 | -0.4 | 94.0 | 94.8 | 95.2 |
|  | AM | 1 | .679 | -2.8 | 0.0 | 0.4 | **93.0** | 95.1 | 95.0 |
|  |  | 2 | .612 | -2.2 | 0.5 | 1.1 | 94.8 | 95.5 | 95.2 |

**Lambda 2 alternative (suggested by a reviewer, not included)**

| I | M | D | P | Bias | | | Coverage | | |
| --- | --- | --- | --- | --- | --- | --- | --- | --- | --- |
|  |  |  |  | *N*=100 | *N*=500 | *N*=2000 | *N*=100 | *N*=500 | *N*=2000 |
| 10 | 2PL | 1 | .582 | **14.7** | **11.9** | **12.3** | **97.4** | **96.4** | **97.5** |
|  |  | 2 | .544 | **14.4** | **11.7** | 9.9 | **97.7** | **96.9** | **97.0** |
|  | AM | 1 | .410 | 5.2 | -0.9 | -3.6 | 95.1 | 94.5 | 95.5 |
|  |  | 2 | .363 | 1.7 | -1.6 | -6.1 | 95.2 | 94.6 | 93.9 |
| 30 | 2PL | 1 | .809 | **32.4** | **34.0** | **34.5** | **98.7** | **99.2** | **99.3** |
|  |  | 2 | .789 | **32.7** | **30.3** | **31.9** | **98.9** | **98.9** | **99.2** |
|  | AM | 1 | .677 | **26.1** | **20.9** | **21.6** | **98.8** | **98.0** | **98.4** |
|  |  | 2 | .639 | 25.4 | 20.3 | 18.9 | **98.0** | **98.4** | **97.9** |

*Note*: I = number of items, M = model, D = Number of dimensions, P = Parameter value, N = Sample size, 2PLM = Two-parameter logistic model. AM = Acceleration model. Bold-faced percentages of scaled bias are greater than 10%. Bold faced coverages percentage lie outside the Agresti-Coull 95% confidence interval [93.95%, 95,88%]. Item parameters that generated the coefficients are the (partially) same across conditions, so conditions within a table are not independent.
